# Induction of Silencing in Plants by High-Pressure Spraying of *In vitro*-Synthesized Small RNAs

**DOI:** 10.3389/fpls.2016.01327

**Published:** 2016-08-30

**Authors:** Athanasios Dalakouras, Michèle Wassenegger, John N. McMillan, Vinitha Cardoza, Ira Maegele, Elena Dadami, Miriam Runne, Gabi Krczal, Michael Wassenegger

**Affiliations:** ^1^AlPlanta-Institute for Plant Research, RLP AgroScience GmbH, Neustadt an der WeinstraßeGermany; ^2^BASF Plant Science, Durham, NCUSA; ^3^Centre for Organismal Studies Heidelberg, University of Heidelberg, HeidelbergGermany

**Keywords:** siRNAs, RNA silencing, *Nicotiana benthamiana*, deep sequencing, spraying, GFP

## Abstract

In this report, we describe a method for the delivery of small interfering RNAs (siRNAs) into plant cells. *In vitro* synthesized siRNAs that were designed to target the coding region of a GREEN FLUORESCENT PROTEIN (GFP) transgene were applied by various methods onto GFP-expressing transgenic *Nicotiana benthamiana* plants to trigger RNA silencing. In contrast to mere siRNA applications, including spraying, syringe injection, and infiltration of siRNAs that all failed to induce RNA silencing, high pressure spraying of siRNAs resulted in efficient local and systemic silencing of the GFP transgene, with comparable efficiency as was achieved with biolistic siRNA introduction. High-pressure spraying of siRNAs with sizes of 21, 22, and 24 nucleotides (nt) led to local GFP silencing. Small RNA deep sequencing revealed that no shearing of siRNAs was detectable by high-pressure spraying. Systemic silencing was basically detected upon spraying of 22 nt siRNAs. Local and systemic silencing developed faster and more extensively upon targeting the apical meristem than spraying of mature leaves.

Since its discovery more than 22 years ago, RNA silencing has been extensively used in crop improvement platforms routinely using dsRNA-expressing transgenes (Tenllado et al., 2004; Eamens et al., 2008; Martinez de Alba et al., 2013). Yet, DCL processing of long dsRNA produces a diverse population of siRNAs and some of them could potentially target additional RNAs resulting in undesirable off-target effects (Voinnet, 2008). Therefore, the introduction of unique siRNA molecules should reduce the risk of off-target effects, at least as long as transitive silencing is not induced. Moreover, the use of transgenes as a source of dsRNA is in most European and other countries under legislative limitations, rendering this strategy problematic for field applications. However, exogenous application of RNA molecules to plants is currently not under legislative limitations. On this premise, Monsanto, among other agricultural companies, puts strong efforts to modify crops by spraying them with polynucleotide molecules, as described in the patent WO 2011/112570 (Sammons et al., 2011). According to this patent, dsRNAs, siRNAs and even single stranded DNA oligonucleotides triggered efficient local and systemic silencing of *Nicotiana benthamiana* endogenes. Given the exciting potential of such a method, we were very much interested to use the method described by Monsanto. Similar approaches to the one described by Monsanto include the application onto plants of crude extracts of bacterially expressed dsRNAs (Tenllado et al., 2003) or of synthetic dsRNAs with carrier peptides (Numata et al., 2014). Besides dsRNAs, siRNAs have been also delivered either to cell cultures through nanosecond pulsed laser-induced stress (Tang et al., 2006) or to protoplasts as conjugated with polymer nanoparticles (Silva et al., 2010). However, with the exception of the Monsanto patent, direct application of siRNAs onto plants for the achievement of local and systemic silencing has not been described before.

Since transgenes are more prone to silencing than endogenes (Vaistij et al., 2002; Koscianska et al., 2005; Christie et al., 2011a,b; Dadami et al., 2013, 2014), and in order to easily monitor the onset and development of silencing, we used the GREEN FLUORESCENT PROTEIN (GFP) -expressing *N. benthamiana* 16C line (Nb-16C; **Figure 1A**) (Voinnet and Baulcombe, 1997). SiRNA duplexes targeting the GFP mRNA were *in vitro*-synthesized and HPLC purified. Similar to endogenous siRNAs, these *in vitro*-synthesized siRNAs contained a 5′ phosphate group, a 3′ methyl group and 2-nt 3′ overhangs (Yang et al., 2006). The siRNA ‘guide’ strand was designed to start with a 5′ uracil residue (U) to be preferentially loaded onto AGO1 (Kim, 2008), and exhibited perfect complementarity with the GFP mRNA. Small RNAs of 22-nt in length and/or 21-nt siRNAs containing asymmetric bulges are proposed to be optimal triggers of transitive and systemic silencing (Chen et al., 2010; Cuperus et al., 2010; Manavella et al., 2012; McHale et al., 2013). Based on these findings, we compared several siRNAs for their potential to trigger RNA silencing. In detail, we used 21-, 22-, and 24-nt long siRNAs containing (siR21asym, siR22asym, and siR24asym) or not containing (siR21, siR22, and siR24) an asymmetric bulge (**Figure 1A**).

**FIGURE 1:**
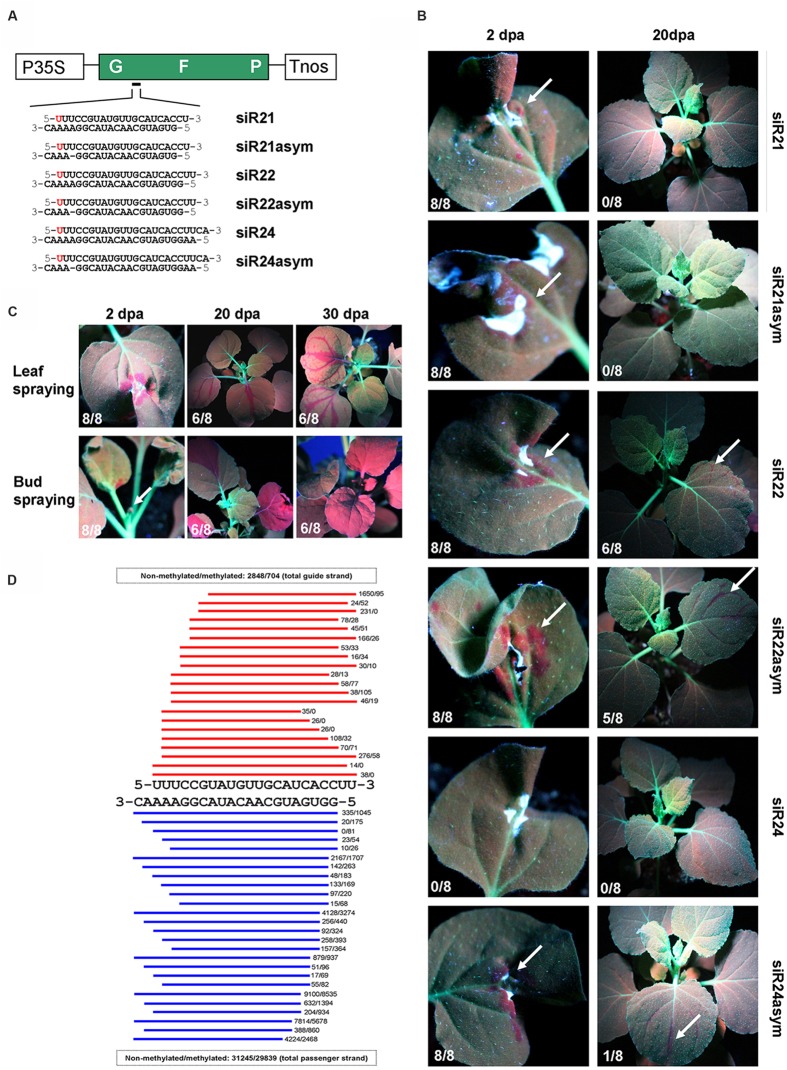
**High-pressure spraying of GFP siRNAs into Nb-16C plants.** **(A)** Schematic representation of the GFP transgene. P35S: 35S promoter of the Cauliflower mosaic virus, GFP: GREEN FLUORESCENT PROTEIN cDNA; Tnos: nopaline synthase terminator. The sequences of 21-, 22-, and 24-nt GFP siRNAs, containing or not containing an asymmetric bulge (‘asym’) are indicated. For all siRNAs, the guide strand starts with 5′-U, indicated in red. **(B)** Monitoring of Nb-16C plants under UV-light 2 and 20 days post application (dpa) of siRNAs by high-pressure spraying, respectively. White arrows indicate regions of silencing. For each experiment eight plants were tested, and the number of silenced plants (X/8) is indicated at the bottom left of each image. **(C)** Monitoring of Nb-16C plants under UV-light 2, 20, and 30 dpa of siR22 by high-pressure spraying, respectively. Spraying was either targeted at the leaf or the bud. White arrows indicate regions of silencing. For each experiment eight plants were tested, and the number of silenced plants (X/8) is indicated at the bottom left of each image. **(D)** Small RNA deep sequencing of Nb-WT plants 2 dpa of methylated and non-methylated siR22 by high-pressure spraying. Red and blue bars represent guide and passenger strands, respectively. The total number of siRNA reads is indicated.

However, we failed to initiate GFP silencing by delivering siRNAs according to the procedure described in the Monsanto patent in which it is stated that ‘any conventional method, e.g., spraying or wiping a solution, emulsion or suspension’ of siRNAs results in the induction of RNA silencing (Sammons et al., 2011). All GFP siRNAs (**Figure 1A**) were applied to Nb-16C either by conventional low-pressure sprayer and/or wiping onto leaves, with or without surfactants (e.g., Silwet L-77) as described in the patent. However, none of these approaches resulted in GFP silencing (Supplementary Figure 1), most probably due to the inability of siRNAs to enter the leaf cells (RNA-uptake). Thus, in order to directly deliver siRNAs into the mesophyll tissue, the GFP siRNAs were infiltrated into Nb-16C leaves. Yet, this approach also did not result in GFP silencing (Supplementary Figure 1), most probably due to the retention of siRNAs in the apoplast. In order to investigate conditions leading to the uptake of exogenously applied RNA molecules into the symplast, we made use of the sensitivity of a viroid infection assay. This assay is based on the fact that only very few infectious viroid RNA molecules need to be introduced into a plant to initiate systemic viroid infection. Using this assay, our data suggested that wounding of tissue is critical for RNA uptake (Supplementary Figure 2). Accordingly, GFP siRNAs that were biolistically introduced into Nb-16C leaf cells efficiently triggered local and systemic GFP silencing (Supplementary Figure 3).

Based on these data, we developed a high-pressure spraying technique that is equally efficient but more convenient and user-friendly method for siRNA delivery than particle bombardment is. Delivery of siRNAs by using a conventional compressor and an air brush pistol resulted in local and systemic RNA silencing (**Figure 1B** and Supplementary Methods). All six GFP siRNAs (**Figure 1A**) were used in high-pressure assays (**Figure 1B**). With the exception of siR24, that exhibited delayed and weak local silencing 10 days post application (dpa), all other GFP siRNAs efficiently induced local silencing 2 dpa (**Figure 1B**). Moreover, siR22, siR22asym, and siR24asym efficiently triggered systemic silencing of the GFP 20 dpa. In contrast, siR21, siR21asym, and siR24 did not induce systemic silencing even 60 dpa. Overall, our data suggested that high-pressure spraying of 22-nt siRNAs, with or without asymmetric bulges, are effective for initiation of local and systemic RNA silencing. The role of 22-nt siRNAs in transitivity and widespread silencing was documented by using artificial micro RNAs (amiRNAs; Chen et al., 2010; Manavella et al., 2012; McHale et al., 2013). However, processing of amiRNAs was shown to be non-uniform resulting in the production of amiRNAs with different sizes from one precursor (Schwab et al., 2006). In contrast, the high-pressure spraying technique enables the introduction of clearly defined siRNAs. Thus, siRNAs of different sizes can be studied for their potential to trigger local, transitive and systemic silencing. Biolistic introduction of siRNAs produced comparable results (Supplementary Figure 3) but biolistic delivery of nucleic acids, including siRNAs may be associated with extensive shearing (O’Brien and Lummis, 2002). Thus, it could be critical to biolistically introduce siRNAs, in particular those with sizes of >21-nt, without size verification of the introduced RNA molecules.

In order to monitor the quality of the input siRNAs used for spraying, deep sequencing of the input siR22 before (siR22-input) and after its passage through the airbrush (siR22-sprayed) was conducted by collecting the sprayed RNA solution into a tube. Essentially identical read numbers of siR22-input (259,546 sense strand, and 208,541 antisense reads), and siR22-sprayed (254,720 sense strand and 208,322 antisense reads) were found. This indicated that the size variants observed in the RNA extracted from sprayed plants by small RNA deep sequencing (**Figure 1D**) derive from *in vivo*-degradation and not from the application method.

High-pressure spaying of siR22 of leaves revealed spotted local silencing patterns and ‘fishnet-like’ systemic silencing patterns (**Figure 1C**). However, spraying of siR22 into apical buds, where apical meristem cells produce the differentiated tissue, led to homogeneous and extended local silencing patterns at emerging leaves and systemic silencing along the entire plant already 20 dpa (**Figure 1C**). Thus, targeting the apical meristem appears to be the most preferable strategy when rapid and extended systemic silencing of the entire plant is desired. Deep sequencing of siRNAs of systemically silenced tissue revealed that GFP siRNAs covering almost the entire GFP coding sequence were produced. With a cut-off of five reads, the total number of reads for 21-nt siRNAs was 41,796 sense and 50,051 antisense, for 22-nt siRNAs 8,208 sense and 8,825 antisense and for 24-nt siRNAs 4,005 sense and 3,247 antisense. This result demonstrated that in the systemic parts of the plant, the response to the systemic silencing signal was associated with transitivity.

In order to investigate the processing of input siRNAs *in planta*, RNA from *N. benthamiana* wild type plants sprayed with methylated and non-methylated siR22 was subjected to small RNA deep sequencing. The data revealed the accumulation of various siRNA sizes ranging from 16-nt (which was the size cut-off) up to 22-nt (**Figure 1D**). They further showed that the presence of a methyl group, proposed to protect small RNAs from uridylation and degradation (Ji and Chen, 2012), did not negate the accumulation of these size variants.

In summary, our data clearly demonstrate that high-pressure spraying of siRNAs can lead to local and systemic silencing in plants. The method itself is not damaging siRNAs. We provide a time-saving and cost-efficient procedure for the introduction of siRNAs which opens the perspective to easily identify molecules efficiently initiating silencing by high-throughput screens. The fact that clearly defined siRNA molecules can be introduced enables the investigation of the potential of siRNA to initiate local and systemic silencing, the study of siRNA movement throughout the entire plant and the analysis of siRNA degradation processes. In addition, silencing may be examined in the absence of transitivity when 21-nt siRNAs not containing an asymmetric bulge (Manavella et al., 2012) are sprayed.

## Author Contributions

AD, Michèle W, VC, IM, ED, and MR performed the experiments. AD, JM, GK, and Michael W conceived the experiments. AD, JM, GK, and Michael W wrote the manuscript.

## Conflict of Interest Statement

The authors declare that the research was conducted in the absence of any commercial or financial relationships that could be construed as a potential conflict of interest.
