# Supplementary material for: Induction of Silencing in Plants by High-Pressure Spraying of In vitro-Synthesized Small RNAs
Source: Front Plant Sci. 2016 Aug 30;7:1327. doi: 10.3389/fpls.2016.01327 (PMC5003833; doi:10.3389/fpls.2016.01327)
Supplement: Supplementary file 1 [file Data_Sheet_1.DOCX]

**SUPPLEMENTAL DATA**

**SUPPLEMENTAL METHODS**

**Application of siRNAs by wiping.**

100 µl of aqueous siRNA solutions (10 µM) were applied as pipette drops to the adaxial surface of Nb-16C leaves.

**Application of siRNAs by infiltration.**

100 µl of aqueous siRNA solutions (10 µM) were infiltrated with a 0.5 ml syringe into the abaxial surface of Nb-16C leaves. Prior to infiltration, a small nick was introduced with a needle to facilitate entry of the solution into mesophyll cells.

**Application of siRNAs by gene gun.**

The Bio-Rad PDS-1000/He biolistic particle delivery system (www.bio-rad.com) was used according to manufacturer's instructions. Essentially, for each plant 1 µg of siRNA was coated onto gold particles and delivered at 900 psi. For the preparation of gold particles (1.0 micron), 10 mg of particles were mixed with 200 µl ethanol (100% v/v), mixed for 1 min, let to settle for 5 min and centrifuged (6,000 g, 10 sec). The supernatant was discarded, particles were washed twice with 100 µl water, washed once with 100 µl glycerol (40% v/v) and finally resuspended in 166 µl glycerol (40% v/v). To 50 µl of this suspension (calculation for 5 plants), 5 µl of siRNA aqueous solution (1 µg/µl) and 20 µl of ice cold spermidine (0.1 M) were added and gently mixed. While mixing, 50 µl CaCl_2_ (2.5 M) were slowly added and mixing was continued for additional 3 min. The solution was placed on ice for 10 min, centrifuged (6,000 g, 10 sec), washed with 100 µl of ice cold ethanol (70% v/v), washed again with 100 µl ice cold ethanol (100% v/v) and finally resuspended in 30 µl ice cold ethanol (100% v/v). For each plant, 6 µl of this suspension was applied on a sterile filter holder, air dried for 10 min and used for biolistic introduction according to the manufacturer's instructions at 900 psi ([www.bio-rad.com](http://www.bio-rad.com)). For each experiment 8 plants at a 4-leaf stage were used.

**Application of siRNAs by high-pressure spraying.**

For each plant, 100 µl of aqueous siRNA solutions (10 µM) were sprayed from a 2-4 cm distance at the abaxial surface of leaves with the CONRAD air brush gun AFC-250A (www.conrad.de) and at a pressure of 7-8 bar provided by the METABO Elektra Beckum Classic 250 compressor (www.metabo.com). For each experiment, 8 plants at a 4-leaf stage were used. For 'leaf spraying' experiments, 2 leaves per plant were used and for 'bud spraying' experiments, the apical meristem of was targeted.

**In vitro siRNA synthesis.**

Duplex siRNAs were *in vitro*-synthesized and HPLC purified by Metabion (www.metabion.com).

**Small RNA deep sequencing.**

Small RNAs were sequenced by GenXPro (www.genxpro.net) as previously described (Dalakouras et al., 2016).

**Reverse transcription polymerase chain reaction (RT-PCR).**

RT-PCR for viroid detection was performed using Superscript III (www.thermofischer.com) according to the manufacturer's instructions. Essentially, 100 ng of total RNA were extracted from apical leaves of inoculated plants 14 days post application. The RNA was amplified using the primers 5´-ACT ACC CGG TGG AAA CAA CT-3´ and 5´-AGG TTT CCC CGG GGA TCC CT-3´.

**Ultraviolet (UV) monitoring**.

For monitoring of GFP fluorescence, the non-UV semiconductor inspection lamps BLAK-RAY (model B 100 AP, UVP, Upland USA) were used.

**References**

**Dalakouras, A., Dadami, E., Wassenegger, M., Krczal, G. and Wassenegger, M.** (2016) RNA-directed DNA methylation efficiency depends on trigger and target sequence identity. *Plant J*.

**SUPPLEMENTAL FIGURES**

**Supplemental Figure 1.**


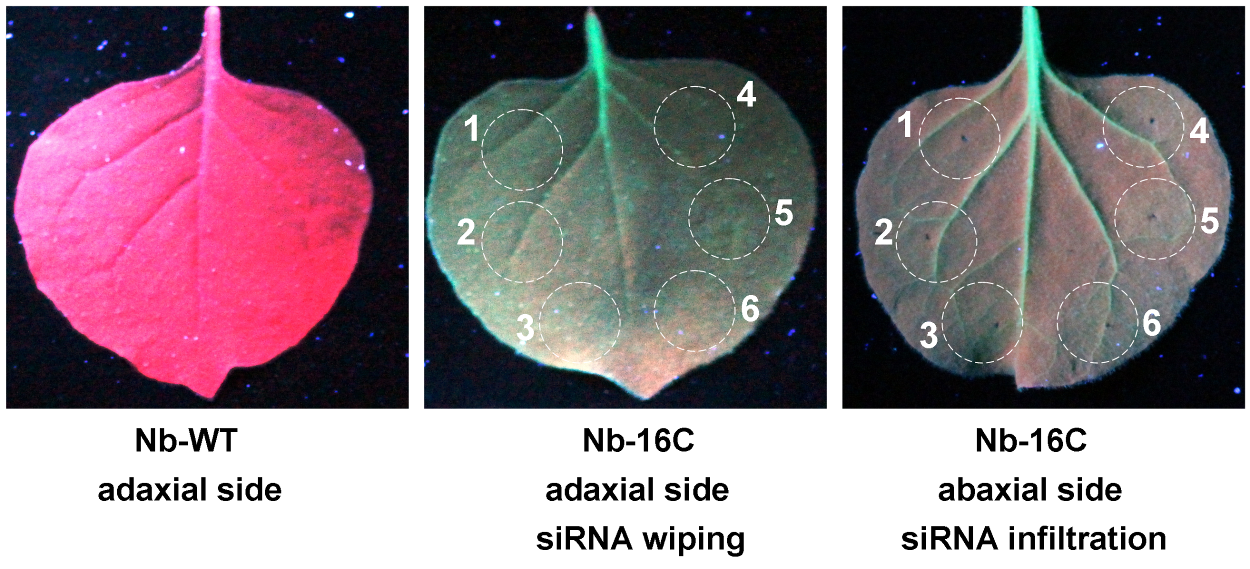


**Supplemental Figure 1.** Application of GFP siRNAs by wiping on the adaxial surface of a Nb-16C leaf (middle panel) and infiltration on the abaxial surface of a Nb-16C leaf (right panel). UV-light monitoring revealed that silencing was not established 8 days post application. The areas of application are indicated with white circles. 1: siR21, 2: siR22, 3: siR24, 4: siR21asym, 5: siR22asym, 6: siR24asym.

**Supplemental Figure 2.**


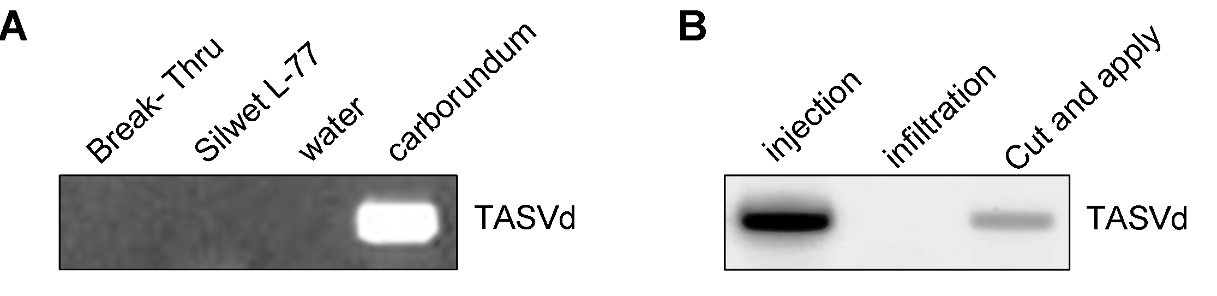


**Supplemental Figure 2.** Infection with Tomato apical stunt viroid (TASVd). **(A)** RT-PCR for the detection of TASVd infection in *N. benthamiana* wild type plants where infectious TASVd RNA (1 µg/µl, 20 µl) was applied in leaves that had been pre-treated with the Break Thru, Silwet L-77, water or carborundum. RT-PCR amplicons were analyzed in 1% agarose gel. **(B)** RT-PCR for the detection of TASVd infection in *N. benthamiana* wild type plants where infectious TASVd RNA (1 µg/µl) was applied by injection into a needle-wounded needle stem, infiltration in leaf mesophyll or as droplets on the stem section. RT-PCR amplicons were analyzed in 1% agarose gel.

**Supplemental Figure 3**


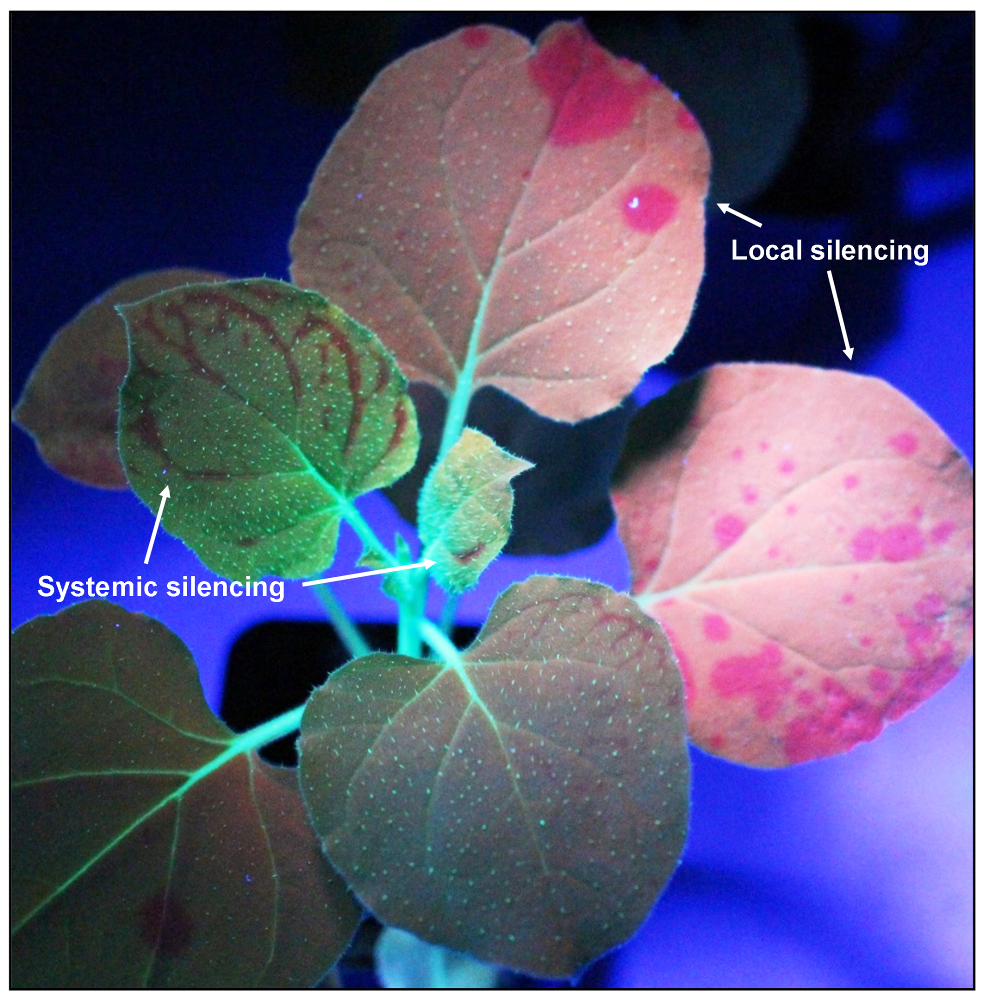


**Supplemental Figure 3.** Biolistic introduction of a 22-nt GFP siRNA containing an asymmetric bulge into Nb-16C. UV-light monitoring15 dpa revealed the establishment of local and systemic silencing.
